# Effects of Intervention Program Prev@cib on Traditional Bullying and Cyberbullying

**DOI:** 10.3390/ijerph16040527

**Published:** 2019-02-13

**Authors:** Jessica Ortega-Barón, Sofía Buelga, Ester Ayllón, Belén Martínez-Ferrer, María-Jesús Cava

**Affiliations:** 1 Faculty of Education, Department of Psychology of Education and Psychobiology, International University of la Rioja (UNIR), Avenida de la Paz, 137, 26006 Logroño, Spain; 2 Faculty of Psychology, Department Social Psychology, University of Valencia, Avda Blasco Ibañez, 21, 46010 Valencia, Spain; sofia.buelga@uv.es (S.B.); Maria.J.Cava@uv.es (M.-J.C.); 3 Faculty of Human Sciences and Education, Department of Psychology and Sociology, University of Zaragoza, Valentín Carderera, 4, 22003 Huesca, Spain; eayllon@unizar.es; 4 Faculty of Social Sciences, Department of Education and Social Psychology, University Pablo Olavide, 41013 Sevilla, Spain; bmarfer2@upo.es

**Keywords:** bullying, cyberbullying, prevention program, Prev@cib, adolescents

## Abstract

Due to the negative consequences of being bullied and the increase in cyberbullying among adolescents, there is a need for evidence-based programs to prevent and intervene in these types of peer violence. The aim of this study was to evaluate the effectiveness of the Prev@cib bullying and cyberbullying program, drawing on three theoretical frameworks: the ecological model, empowerment theory, and the model of personal and social responsibility. The Prev@cib program was evaluated using a repeated-measures pre-post-test design with an experimental group and a control group. The sample consisted of 660 adolescents between 12 and 17 years old (*M* = 13.58, *SD* = 1.26), randomly assigned to the experimental and control groups. Repeated-measures ANOVA of pre-post-test scores were conducted. Results showed a significant decrease in bullying and victimization and cyberbullying and cybervictimization in the experimental group, compared to the control group, indicating that the Prev@cib program is effective in reducing bullying and cyberbullying. Taking into account the harmful effects of these types of violence, the results have important implications in the prevention of these behaviors because they provide scientific evidence of the program’s effectiveness.

## 1. Introduction

### 1.1. Bullying and Cyberbullying

Information and communication technologies have many advantages for adolescents, allowing them to have unlimited access to all types of information and fostering interactions with peers at any place and time [1,2]. However, these tools have enabled new forms of violence, such as cyberbullying, to emerge [3,4]. Cyberbullying is defined as intentional and aggressive behavior repeated frequently over a period of time through the use (by an individual or group) of electronic devices against a victim who cannot easily defend him/herself [5]. 

Bullying and cyberbullying have some features in common, such as the imbalance of power between the victim and the aggressor and the intentionality and repetition of the violent behavior [6,7]. These similarities lead some authors to consider cyberbullying to be a modality of traditional bullying [8,9,10]. In fact, numerous studies have found continuity between school bullying and cyberbullying [11,12,13], justified by the connection between the offline and online environments [14]. Thus, the study by Hinduja & Patchin (2010) [15] showed that over 60% of adolescents were involved in both forms of bullying. Therefore, it is common for aggressors emerging in the classroom to continue this behavior elsewhere through the smartphone and Internet.

This continued bullying in offline and online settings seems to produce a high level of psychological distress and behavior problems in the victim [10,16], as well as strong feelings of anxiety, depression, fear, nervousness, irritability, somatizations, sleep disorders, and concentration difficulties [17,18]. Victims of bullying and cyberbullying are more likely to have suicidal ideations and make suicide attempts [15,19], which also negatively affect the family and social environment [20,21,22]. Bullies and cyberbullies also experience negative consequences that can persist in adult life. Different studies have shown that bullying and/or cyberbullying aggressors have greater depression symptoms, break the rules more, consume more drugs, have a more negative attitude toward authority figures, and participate more in violent and criminal behaviors in other areas of their lives [23,24]. Additionally, increased awareness and reporting of mental health problems that may stem from bullying justify the importance of designing and implementing anti-bullying programs during adolescence [25].

### 1.2. Intervention Programs in Bullying and Cyberbullying

Due to the social concern about bullying and cyberbullying, numerous programs have been developed to prevent and intervene in these two modalities of peer violence [26,27,28]. Most of these programs have focused specifically on bullying or cyberbullying. For example, the Olweus Bullying Prevention Program [29] and Social Skills Training [30] are designed to reduce and prevent bullying and victimization. In addition, some antibullying interventions have included other psychosocial variables, such as empathy, self-esteem, or sensitivity towards the victims [31,32], which are understood as resources that prevent and reduce school bullying. Other antibullying programs have been designed to reduce bullying, but were also found to be useful for reducing cyberbullying, such as the Kiva program [33] and the ViSC Social Competence Program [34]. 

In general, previous meta-analyses and systematic reviews have provided little scientific evidence about the effectiveness of bullying and cyberbullying interventions [35,36,37]. In fact, compared to bullying programs, few programs that focus specifically on cyberbullying have been experimentally assessed. Some programs that have shown positive effects in preventing and reducing cyberbullying are the Tabby Improved Program [38] and the Cyber Friendly Schools Project [39]. The majority of the programs focused on cyberbullying work on both types of bullying, for example, The Media Heroes Cyberbullying Prevention Program [40] and the Cyberprogram 2.0 [41]. The authors of these programs consider cyberbullying and bullying to be closely linked, making it crucial to address these two problems together.

In sum, due to the great social relevance that cyberbullying is acquiring worldwide, it is fundamental to develop anti-cyber(bullying) programs that are clearly grounded in theoretical frameworks and experimentally tested.

### 1.3. Prev@cib Program

Prev@cib is based on three theoretical frameworks: the ecological model [42], empowerment theory [43], and the personal and social responsibility model by Hellison (1995) [44]. First, regarding the ecological model, several individual, microsocial, and contextual risk and protection factors related to traditional bullying and cyberbullying were taken into account [42]. Although the Prev@cib program is focused on students, we conducted a course to train teachers in how to implement the program. In this context, we took the teachers’ opinions and comments into account throughout the implementation of the Prev@cib program. Second, in order to offer adolescents tools and resources to use when facing this type of problem, this program also draws on empowerment theory [43]. According to this theory, individual, group, and community resources are strengthened as a basic strategy to allow adolescents to control their lives in both the virtual and school environments. In this regard, the Prev@cib program offers different resources and coping strategies to better deal with the problem of bullying and cyberbullying. Third, this program is also based on the personal and social responsibility model by Hellison (1995) [44], which argues that responsible behaviors can be taught and generalized to other contexts in life. This theory is used to encourage the idea of shared responsibility in the problems of bullying and cyberbullying, in order to achieve adolescents’ greater involvement in their prevention and reduction. Based on this theory, in Module 3, the Prev@cib program focuses on the importance of involving all the students in solving and impeding peer bullying and cyberbullying.

Grounded in this theoretical foundation, the Prev@cib consists of 10 one-hour sessions distributed in three modules: information, awareness, and involvement (Table 1).

- Module 1. Information about risk and prevention factors in the bullying and cyberbullying problem. This module also includes information about sexting and grooming. In fact, some studies have found evidence of a relationship between cyberbullying and sexting and cybergrooming [16,45]. The module consists of four sessions designed to provide adolescents with information about the characteristics, types, and risk factors associated with these problems. In this way, the adolescents increase their awareness and detection of the existing dangers, especially in the virtual world. They are also taught strategies to protect themselves on the Internet and avoid becoming involved in potential cyberbullying problems.
- Module 2. Awareness and sensitization about cyberbullying. This consists of two sessions designed to make the participants aware of and sensitive to the harm and negative consequences of peer violence, both in school and through technologies. It is important for adolescents to understand the harmful consequences of school and cybernetic violence, in order to foster changes at cognitive, behavioral, and attitudinal levels and prevent and reduce this peer abuse at school and online.
- Module 3. Involvement in and commitment to prevention and intervention in cyberbullying. This module is composed of four sessions designed to encourage students’ involvement in and commitment to preventing and acting on this problem. In this regard, an emphasis is placed on the role of all the students in stopping and preventing the appearance and continuance of bullying. Thus, in the classroom, a climate of respect is fostered, so that no type of violence is tolerated among the adolescents in the school or virtual environment.

In addition to these 10 intervention sessions, the program contains two other one-hour sessions to experimentally evaluate the effects of the program (pre-test and post-test).

To implement this program, the recommendations of Garaigordobil and Martínez-Valderrey (2014) [41] were followed, regarding: inter-session constancy (the same interval between one session and another); time-space constancy (all the sessions held at the same time and in the same classroom, whenever possible); constancy in the person who implements the program (the same person administers all the sessions); and constancy in the session structure (same session structure, even though the contents and activities carried out may differ).

Taking into account the need for evaluations of programs focused on bullying and cyberbullying prevention, the main aim of the present study is to evaluate and test the effectiveness of the Prev@cib program on (cyber)bullying and (cyber)victimization among adolescent students. Specifically, the hypotheses are: (1) after the program, the intervention group will obtain lower scores on violent school behavior than the control group; (2) the intervention group will obtain lower scores on school victimization than the control group; (3) the intervention group will obtain lower scores on cyberbullying than the control group; and (4) the intervention group will obtain lower scores on cybervictimization than the control group.

## 2. Materials and Methods

### 2.1. Study Design and Participants

To evaluate the effects of the Prev@cib program, a repeated-measures (pre-test and post-test) quasi-experimental design was used with an experimental group and a control group. Initially, the sample was composed of 692 adolescents. Thirty-two participants were eliminated (4.63% of the sample) because they did not correctly fill out the questionnaires or because they missed more than one session of the Prev@cib program.

The final participants in the Prev@cib program were 660 adolescents (53.2% girls and 46.8% boys) between 12 and 17 years old (*M* = 13.58, *SD* = 1.26). The participants belonged to 35 classes from four high schools (compulsory secondary education) in Valencia (Spain). Of them, 28.8 percent were in 7th grade, 32 percent were in 8th grade, 21.5 percent were 9th grade, and 17.5 percent were in 10th grade. 

As Table 2 shows, 434 students (24 classes) participated in the experimental group, and 236 students (11 classes) in the control group. The average number of students per class was 23. For gender, age, and grade in school, no significant differences were found between experimental and control participants, so that both groups were similar in terms of age (*t* = −2.10; *p* = 0.361), gender (χ^2^(1) = 0.33; *p* = 0.568), and grade in school (χ^2^(1) = 0.01; *p* = 0.919).

### 2.2. Procedure

Various informative meetings were held with the selected schools to explain the objectives and methodology of the Prev@cib program. The high schools were selected through non-probability convenience sampling based on their accessibility and previous interest in participating in this study. After obtaining parent permission and authorization, the researchers randomly assigned the adolescents to one of two groups: (1) an experimental group, where the Prev@cib program was implemented; (2) a control group, where the program was not implemented.

To evaluate the short-term effects of this pilot program, all the adolescents (experimental and control group) filled out a structured pen-and-paper questionnaire in their classrooms. Under the supervision of a least one of the researchers, this self-report questionnaire took approximately one hour to complete. When the questionnaires were administered, the adolescents were told that their participation would be voluntary and anonymous. 

The Prev@cib program, which lasted 9 months, was implemented in the experimental group during their homeroom schedule. A pre-test was carried out in September 2016; a post-test in May 2017. The intervention took place from October through April 2017. The experimental and control groups filled out a battery of instruments in the pre and post-test phases. The Prev@cib program was administered by 13 teachers and four researchers previously trained by one of the investigators in this study. 

Parental consent for participation was received from all participants. Furthermore, all the adolescents gave their informed consent before they participated in this study. This study followed the ethical values established in the 1964 Declaration of Helsinki and its later amendments, and the UNESCO Universal Declaration of Human Rights. In addition, all the procedures performed in the study were approved by the Ethics Committee of the University of Valencia, Spain (Project identification code: H1456762885511).

### 2.3. Measurement Variables and Instruments

Scale of Peer Victimization at School [46,47]. This instrument is composed of 12 items that evaluate the degree of victimization at school in the past school year (e.g., “A classmate hit or punched me” or “A classmate separated me from my group of friends”). Responses are given on a Likert-type scale ranging from 1 to 5 (never, only once, a few times in the past month, many times in the past month, and this happens to me quite often). Cronbach’s alphas for this scale in this study were 0.88 (pre-test) and 0.90 (post-test).

Scale of School Aggression [48,49]. This Likert-type scale is composed of 12 items with a response range from 1 to 5 (never, seldom, sometimes, often, and always). This scale evaluates aggressive behaviors toward peers in the school context in the past 12 months (e.g., “I am someone who hits, kicks, and punches others”). Cronbach’s alphas for this scale in this study were 0.72 (pre-test) and 0.79 (post-test).

Scale of Victimization through the Cell Phone and Internet [50]. The CYBVIC scale is composed of 15 items that measure the adolescent’s experience as a victim of cyberbullying through the cell phone or Internet in the past 12 months (e.g., “I have been insulted or ridiculed through social networks, Internet, or cell phone”). From the victim’s perspective, this scale measures cybernetic behaviors of harassment, persecution, belittlement, invasion of privacy, social exclusion, and identity theft. The items were responded to using a Likert-type scale with five response options (never, seldom, sometimes, often, quite often). Cronbach’s alphas for this scale in this study were 0.88 (pre-test) and 0.89 (post-test).

Scale of Aggression through the Cell phone and Internet [50]. The CYB-AGRESS scale is composed of 15 items that measure the frequency with which the respondent has participated in aggressive behaviors through new technologies in the past 12 months (e.g., “I have insulted or made fun of someone through social networks, Internet, or cell phone”). From the aggressor’s perspective, the scale measures cybernetic behaviors of harassment, persecution, belittlement, invasion of privacy, social exclusion, and identity theft. The items are answered on a Likert-type scale with five response options (never, seldom, sometimes, often, and a lot). Cronbach’s alphas for this scale in this study were 0.80 (pre-test) and 0.90 (post-test). 

Prior to administration, the definitions of bullying and cyberbullying were provided for all scales, and adolescents responded with this type of behavior in mind. In addition, on these scales, the adolescents were asked about the duration of the episodes and the frequency and persistence of bullying and cyberbullying.

### 2.4. Statistical Analysis

Data analysis was carried out using the SPSS statistical package (version 22, SPSS Inc., Chicago, IL, USA). To evaluate the effects of the program on each of the study variables, several 2 x 2 mixed factorial ANOVAs were used, with a between-subjects factor (experimental group and control group) and a within-subjects factor (before and after the program: pre-test and post-test). The use of this analysis is recommended when the groups selected are natural and not equal in the initial situation [51]. The interaction term in the mixed factorial ANOVA describes the effect of the program and is equivalent to t-tests on difference scores (post-test pre-test). The eta-square (η^2^) value is used as an indicator of the size of the effect. Cohen (1988) suggested that η^2^ ≤ 0.06 can be considered a ‘small’ effect size, 0.07 ≤ η^2^ ≤ 0.14 represents a ‘medium’ effect size, and >0.14 is a ‘large’ effect size [52].

## 3. Results

### 3.1. Effects of Intervention Program Prev@cib on Traditional Bullying

**Figure 1:**
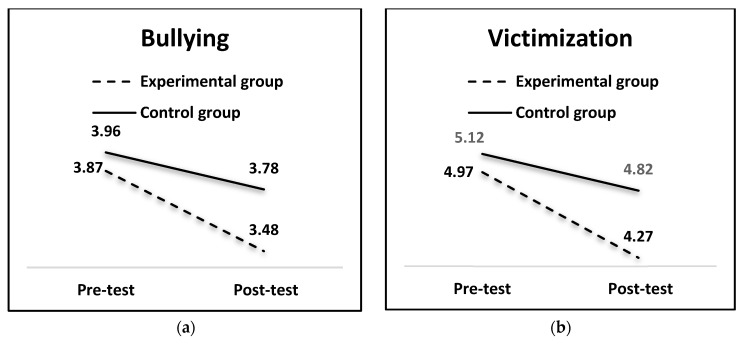
Means obtained by the groups (experimental and control) on bullying (**a**) and victimization (**b**).

Regarding bullying, a significant group x time interaction effect was found *F*(1, 658) = 6.67, *p* < 0.01, with a medium effect size, *η*^2^ = 0.09. As Table 3 and Figure 1 show, although bullying decreased in both the experimental and control groups, this decrease was significantly more pronounced in the experimental group. The same pattern was obtained for victimization, *F*(1, 658) = 7.80, *p* < 0.01, with a medium effect size, *η*^2^ = 0.10; victimization at post-test was lower than at pre-test, especially in the experimental group.

### 3.2. Effects of Prev@cib Intervention Program on Cyberbullying

**Figure 2:**
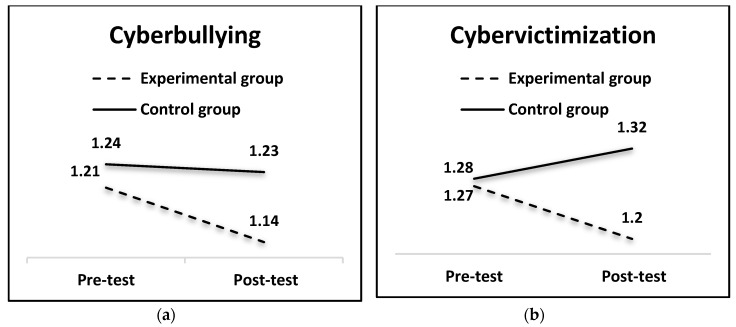
Means obtained by the groups (experimental and control) on cyberbullying (**a**) and cybervictimization (**b**).

For cyberbullying, results yielded a significant group ×time effect, F (1, 658) = 7.03, *p* < 0.01, with a small effect size η^2^ = 0.05. Findings indicated that cyberbullying remained stable in the control group, whereas it decreased in the experimental group (see Table 4 and Figure 2). A significant group × time effect was also obtained for cybervictimization F (1, 658) = 11.63; *p* < 0.001, with a small effect size, η^2^ = 0.04. As Table 4 and Figure 2 show, cybervictimization increased slightly in the control group, whereas it decreased in the experimental group.

## 4. Discussion

The main objective of this study was to experimentally assess the effects of the Prev@cib Program. Specifically, the short-term impact of the Prev@cib Program was shown on bullying and cyberbullying perpetration and victimization among adolescents.

The Prev@cib program is based on three theories that support the contents of the program. This theoretical framework provides a solid and rigorous basis for this novel and necessary proposal in this area of research. Thus, the Prev@cib program is based on the ecological model [42] because it emphasizes the protector factors and personal, microsocial, and contextual risk factors associated with bullying and cyberbullying. Second, it also contains principles and theoretical constructs of empowerment theory [43], related to resources and strategies for controlling and exercising self-determination over one’s life. In this case, these resources help adolescents to improve life at school with peers and avoid poor use of new technologies. For example, the program contains activities about cybernetic security measures or the legal consequences of violence perpetration, both “face to face” and virtual. Third, the Prev@cib program is based on the model of personal and social responsibility [44]. Thus, it addresses the need for adolescents to accept shared responsibility in bullying and cyberbullying problems, in order to involve them in preventing and reducing these forms of violence. Thus, adolescents are made aware that, through their silence, they contribute to maintaining the situation of peer abuse. 

The findings emphasize the effectiveness of the Prev@cib intervention program for both bullying and cyberbullying. Specifically, in the experimental group, a reduction was observed in the involvement in bullying and cyberbullying as aggressors and victims, compared to the control group. This decrease in bullying and cyberbullying in the experimental group could be explained by the variety and suitability of the contents. The positive results of Prev@cib are consistent with other programs that have also shown their experimental effectiveness [27,28,37]. 

Consequently, all the hypotheses proposed in our study were confirmed. Regarding the hypotheses related to “face to face” bullying, other programs also obtained positive effects in reducing victimization and perpetration of this type of violence, for example, the Olweus Bullying Prevention Program [29] or the ViSC Social Competence Program [34]. Previous studies emphasized the importance of the school environment in reducing the rates of bullying [53,54]. In this regard, the Prev@cib program was designed to reduce and prevent aggressiveness through activities involving cooperative learning, understanding, and shared responsibility in the school. Thus, the school climate is fostered as a protector factor, rather than a risk factor, in the manifestation of aggressive behaviors. This intervention is fundamental because adolescents who exhibit aggressive behaviors toward their classmates have been found to present other violent behaviors outside the classroom [24,55]. The Cyberprogram 2.0 also shares this focus by emphasizing conflict resolution strategies in the school context in order to reduce impulsive and premeditated aggressiveness in other social settings [41]. Future research should study the effects of the Prev@cib program on reducing these two types of aggressiveness, premeditated and impulsive, in order to more closely examine these expressions of violence toward peers.

In addition, the Prev@cib Program significantly reduced school victimization. The implications of these positive results are important for the victims of school bullying, who usually experience feelings of anxiety, depression, psycho-somatizations, suicidal ideation, and problems in school as a result of the continued abuse [17,56]. These negative effects of bullying increase even more when the victim is not only bullied in the school context, but also through new technologies [20,57]. Along these lines, in the Media Heroes Cyberbullying Prevention Program [40], the most positive effects of the program were obtained in victims who experienced bullying and cyberbullying at the same time, by significantly reducing their psychological distress. It is important for adolescents to be aware of the seriousness of the problem and become sensitive to the suffering of the victims in order to eradicate or reduce these types of violence [58,59]. From this perspective, the Prev@cib program specifically addresses this question in an awareness module through, for example, the activity “What if you were the victim?”

Moreover, other main objectives of the Prev@cib program related to the last two study hypotheses also showed effective results in reducing cyberbullying. Whereas in the intervention group, cyberbullying decreased in the post-test phase, in the control group, these behaviors did not decline, and instead remained constant over time. These results are congruent with those from other programs that obtained positive effects on reducing cyberaggression, such as The Brief Internet Cyberbullying Prevention Program [60] or Tabby Improved program [38]. An interesting question related to cyberbullying behaviors involves victims of traditional bullying who use the Internet to get revenge. Indeed, the impunity offered by the anonymity of the Internet has led some bullying victims to view the virtual space as the ideal way to punish their aggressors [61]. The Arizona program focuses on this issue, producing a reduction in revenge by victims after the intervention [62]. One of the future challenges of intervention programs, including ours, is to try to eradicate these types of vengeful behaviors where bullying victims become cyberbullies in the virtual space. 

In the case of cybervictimization, the results of the Prev@cib program also confirmed the hypothesis of a decline in cybervictimization in the intervention group, compared to their baseline and the control group. Thus, whereas cybervictimization decreased in the intervention group, it increased in the control group. Along these lines, other programs such as the Cyber Friendly Schools Project [39] or Surf-Fair [63] also obtained positive effects on reducing cybervictimization in the participants. Taking into account that (cyber)victimization causes several types of present and future psychosocial damage in targets of this type of violence [64], it is important to join forces to protect our children and adolescents. Furthermore, as other studies have suggested, if we do not intervene in this problem, we run the risk of cyberbullying becoming normalized in adolescence, thus affecting adolescents’ wellbeing and development, as well as peaceful co-existence in high schools [15,65].

In sum, the Prev@cib program has been shown to have positive effects on reducing bullying and cyberbullying, and also aggression and victimization. However, the effect size of the program is smaller for cyberbullying than bullying. This result is consistent with those obtained by different authors [66]. It has been pointed out that cyberbullying program effects are often limited to increasing Internet safety knowledge [67]. Future research interventions should provide some online sessions to foster interpersonal connectivity through the Internet. 

The results support the idea that adolescents involved in traditional bullying are also usually involved in cyberbullying [11,68]. Based on this assumption of continuity between traditional bullying and cyberbullying, the Prev@cib program, like programs by other researchers, includes activities to address both problems with the participants [27,36]. 

This study has some limitations that should be taken into account when interpreting the conclusions of this study. The first limitation is related to the representativity of the sample. Although the sample of participants is large because it is a classroom intervention, the generalization of the results to the adolescent population must be carried out with caution. Future studies could implement the Prev@cib program in other samples of adolescents from other countries. A longitudinal study should also be carried out to test the stability of the long-term changes observed in the intervention group. It would also be interesting to include other variables, such as school climate, to observe how it changes during the program. In this regard, some authors suggested that cyberbullying victims do not perceive the teacher as a source of authority who helps to solve their bullying problems with peers [24,58]. This lack of confidence in teachers indicates not only that they should be included in intervention programs, but also that teachers’ support should be evaluated after the program. In addition, another limitation is the small effect size of the Prev@cib program on cyberbullying. According to some meta-analyses and review studies [27,36], in general, the effect sizes of bullying and cyberbullying intervention programs are usually small. Another limitation is that students are nested within 35 classes from four schools, which may lead to low independence in the data. Although groups were randomly assigned to ensure their equivalence [69], it was impractical to randomize participants at an individual level, due to the risk of social threats to validity [70]. Future research should take this limitation into account by considering class-level and school-level analysis and conducting a multilevel or hierarchical analysis. Finally, another limitation of this manuscript could be the use of self-reports as the only assessment instruments in the study. Even so, previous studies highlight the acceptable reliability and validity levels of adolescent self-reports to measure risk behaviors [71,72].

In spite of the limitations, this study provides a novel program to prevent bullying and cyberbullying in adolescence, based on scientific evidence. Prev@cib is a theoretically-based program that has been shown to have positive effects on reducing bullying and cyberbullying perpetration and victimization.

## 5. Conclusions

This study presents the experimental validation of the effects of the Prev@cib program, which has the objective of preventing and reducing bullying and cyberbullying among adolescents. Specifically, this program is based on three theoretical frameworks: the ecological model, empowerment theory, and the personal and social responsibility model. Regarding the contents, the Prev@cib program consists of ten sessions distributed in three modules: (1) Information; (2) Awareness and sensitization; and (3) Involvement and commitment. To evaluate the effectiveness of the Prev@cib program, a quasi-experimental repeated-measures pre-test and post-test design was used with an experimental group and a control group. 

The results of the present study showed the efficacy of the Prev@cib program. The program had positive effects on reducing bullying in the participants. Specifically, the findings show that in the experimental group, compared to the control group, school perpetration and victimization behaviors declined significantly. These positive effects were also observed for cyberbullying in the experimental group.

In summary, our results present scientific evidence that the Prev@cib program is effective in reducing and preventing bullying and cyberbullying in the adolescent population.

## Figures and Tables

**Table 1:** Sessions of the Prev@cib program.

	Modules	Sessions
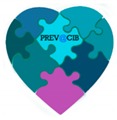	Module 1. Information	Session 1. My life is a display window
		Session 2. Bullying and cyberbullying
		Session 3. Sexting and grooming
		Session 4. Cyber-protection
	Module 2. Awareness	Session 5. Consequences and we are all responsible
		Session 6. What if you were the victim?
	Module 3. Involvement	Session 7. What to do when faced with bullying?
		Session 8. (Cyber)helpers
		Session 9. I like myself, I like you
		Session 10. No more bullying

**Table 2:** Sample characteristics and group differences by condition: frequency and percentage.

Variables	Experimental Group (n = 434)	Control Group (n = 236)	*p*
**Age M (DT)**	13.50 (1.29)	13.72 (1.21)	0.361
**Sex**			0.568
Boys	229 (34.7%)	122 (18.5%)	
Girls	195 (29.5%)	114 (17.3%)	
**Grade in secondary education**			0.919
Grade 7	136 (20.6%)	54 (8.2%)	
Grade 8	121 (18.3%)	92 (13.8%)	
Grade 9	88 (13.4%)	52 (7.9%)	
Grade 10	77 (11.7%)	40 (6.1%)	

Note: Age (*t* test), gender, and grade in school (Chi squared).

**Table 3:** Between-group effects and repeated-measures analysis of variance (ANOVA 2 × 2) in bullying.

Variables	*M* (*DT*)			*F (p)*			η^2^
	Group	Pre-test	Post-test	Time Effect	Group Effect	Interaction Effect	
**Bullying**	Experimental	3.87 (0.92)	3.48 (1.07)				
	Control	3.96 (0.96)	3.78 (0.93)	48.95 ***	8.40 **	6.67 *	0.09
**Victimization (bullying)**	Experimental	4.97 (1.89)	4.27 (1.67)				
	Control	5.12 (2.02)	4.82 (1.90)	36.26 ***	7.80 **	5.75 *	0.10

Note: η^2^ = Eta squared effect size; 0.07 ≤ η^2^ ≤ 0.14 = medium size; * *p* < 0.05; ** *p* < 0.01; *** *p* < 0.001.

**Table 4:** Between-group effects and repeated-measures analysis of variance (ANOVA 2 × 2) in cyberbullying.

Variables	*M* (*DT*)			*F (p)*			η^2^
	Group	Pre-test	Post-test	Time Effect	Group Effect	Interaction Effect	
**Cyberbullying**	Experimental	1.21 (0.28)	1.14 (0.32)				
	Control	1.24 (0.34)	1.23 (0.41)	7.39 **	7.03 **	4.67 *	0.05
**Cyber Victimization**	Experimental	1.27 (0.41)	1.20 (0.32)				
	Control	1.28 (0.39)	1.32 (0.47)	1.16	6.38 *	11.63 ***	0.04

Note: η^2^ = Eta squared effect size; η^2^ ≤ 0.06 = small size; * *p* < 0.05; ** *p* < 0.01; *** *p* < 0.001.
